# COVID-19 infection rates and mitigation strategies in orthodontic practices

**DOI:** 10.1186/s12903-022-02705-1

**Published:** 2023-01-07

**Authors:** Peter M Durbin, Grace Viana, Veerasathpurush Allareddy, Budi Kusnoto, Sriram Ravindran, Shrihari Kadkol, Phimon Atsawasuwan

**Affiliations:** 1 Department of Orthodontics, College of Dentistry, University of Illinois Chicago, Chicago, IL United States of America; 2 Department of Oral Biology, College of Dentistry, University of Illinois Chicago, Chicago, IL United States of America; 3 Department of Pathology, College of Medicine, University of Illinois Chicago, Chicago, IL United States of America

**Keywords:** Orthodontic providers, COVID-19, COVID-19 vaccine, Infection control

## Abstract

**Background:**

COVID-19 has impacted and increased risks for all populations, including orthodontic patients and providers. It also changes the practice management and infection control landscape in the practices. This study aimed to investigate the COVID-19 infection and vaccination status of orthodontic providers and mitigation approaches in orthodontic practices in the United States during 2021.

**Methods:**

A validated 50-question research electronic data capture (REDCap) browser-based questionnaire was distributed to 12,393 orthodontists and pediatric dentists who reported actively providing orthodontic treatment. Questions were designed to collect demographic data of respondents, evaluate the COVID-19 mitigation approaches, and evaluate the history of COVID-19 infection and vaccination status of the orthodontic providers. Associations of demographic and the COVID-19 mitigation approaches were assessed using chi-square tests at the significance level of 0.05.

**Results:**

Four hundred fifty-seven returned the survey (response rate 3.69%) for analysis. Most respondents were vaccinated, and increased infection control measures in response to the pandemic. Half of the respondents practiced teledentistry and switched to digital impression systems. Two-thirds reported difficulties in attaining PPEs due to the increased cost and scarcity of PPEs. About 6% of respondents reported a history of COVID-19 infection, and 68.9% of their staff had COVID-19 infection. Statistically significant associations were found between increased practice experience with difficulties in acquiring PPE (*p* = .010). There were no significant associations between races of respondents, geographic location, and years of practicing when cross-tabulated with vaccination status or COVID-19 infection rate (*p* > .05).

**Conclusion:**

Increased infection control strategies were employed in almost all orthodontic practices in addition to existing universal precaution. Most of the orthodontic providers and their staff members were vaccinated. While staff’s infection rates were an issue, doctors’ infection rates remained low.

## Background

A novel coronavirus was discovered in Wuhan, China, at the end of 2019 [1]. In February 2020, the World Health Organization designated the virus as severe acute respiratory syndrome coronavirus 2 (SARS-CoV-2), which causes Coronavirus disease 2019 (COVID-19) [2]. The SARS-CoV-2 virus is mainly transmitted through exposure to infectious respiratory fluids, especially the inhaling very fine respiratory droplets and aerosols [3] and can occur in asymptomatic, presymptomatic, and symptomatic stages of infection [4]. This nature of transmission puts dental healthcare providers at increased risk of infection, as orthodontic providers regularly perform aerosol-producing procedures [5]. The most significant risk of transmission via inhalation is within three to six feet of an infectious source [3] while another mean of possible in-office transmission is touching oral/nasal mucous membranes with hands contaminated with exhaled respiratory fluids or contaminated surfaces [3, 6]. With the spread of new variants, there is concern that symptoms may worsen as the virus mutates and lead to the next surge of a pandemic [7]. COVID-19 vaccines have been shown as one approach to control the development of virus mutation and to contraction of COVID-19 effectively and significantly reducing severe disease, hospitalization, and death [8]. The Centers for Disease Control and Prevention (CDC) has launched the guidelines to implement COVID-19 mitigation for dental procedures to prevent in-office transmission [9]. A study gauging COVID-19 positivity rates in dental hygienists in the United States found that 3.1% had tested positive or been diagnosed with COVID-19 [10], while the rate in general dentists was found to be lower (0.91%) [11] with 2.6% in a 6-month longitudinal follow-up study [12]. COVID-19 also affected the mental health of dental healthcare workers as fluctuated anxiety and depression [13, 14]. Few studies reported the positivity rates of patients seeking dental treatments including emergency, pediatric and orthodontic treatments ranging from 0.027 to 6.7% [15–19]. Due to its high transmitted nature, COVID-19 leads to the report of 99.7% of dentists enhancing PPE protocols to mitigate the COVID-19 transmission [11]. An online questionnaire study in orthodontists to investigate the source of information for COVID-19 in 2020 demonstrated that their most accessed information sources were professional association websites (> 70%) and online news sources (61%) which the state or local dental associations (53%) and the American Association of Orthodontists (50%) were reported as the most valuable sources of information [20]. Though the guidelines to mitigate COVID-19 transmission in dental practices exist, there are no reports on how the actual approaches were implemented especially certain groups such as orthodontic providers, which the nature of their practices was different from other types of dental practices.

## Methods

This study aimed to investigate orthodontic providers’ COVID-19 infection and vaccination rates and mitigation approaches in orthodontic practices in the United States in 2021.

### Participants

The voluntary survey was disseminated to 4,414 active members of the American Association of Orthodontists (AAO) and all 7,887 active members of the American Academy of Pediatric Dentistry (AAPD), and all 92 members of the Angle Midwest Society. The survey was performed from January 1st, 2021 to December 31st, 2021. To maximize the resulting number of respondents no sampling scheme was adopted. Due to the descriptive nature of the survey, no formal prospective sample size calculations applicable to hypotheses testing or error rates were attempted. We distributed the questionnaires to all members of the American Association of Orthodontists and the American Academy of Pediatric Dentistry and used the inclusion and exclusion criteria to determine the sample numbers for the data analysis.

### Ethical consideration

This survey study was granted exemption from the University of Illinois Chicago Institutional Review Board (#2020 − 1469). All participants joined the study voluntarily and anonymously and the informed consent was stated when the participants logged in for the questionnaires.

### Questionnaire design

**Fig. 1:**
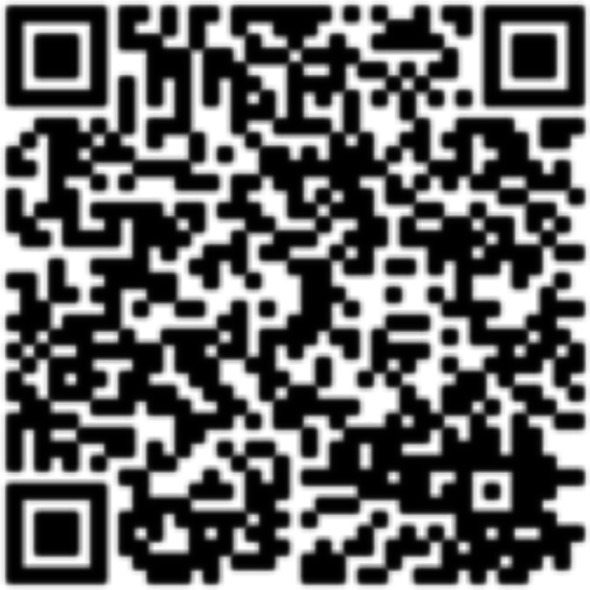
QR code linked to the set of questionnaires. The QR code was distributed to the participant via electronic mail

The questionnaires were generated using REDCap (Research Electronic Data Capture) platform. A draft questionnaire was developed and validated with a panel of 40 experienced orthodontists to evaluate the questions and provide input regarding the validity, length, sequence, and relevance of the questions. The questions were distributed to 40 orthodontists in private practices and academic institutes to evaluate the validity of the questions and feedback. These processes were conducted to establish the solid structure of the content and face validity of the questions and to ensure the answers render the understanding of the COVID-19 mitigation approaches and the nature of infection rate and vaccination rates in the orthodontic providers. The questions were modified according to the expert panel’s feedback and the fluidity of federal COVID-19 restrictions. The final 50-item question survey consisted of yes/no options, dropdown choices, multiple-choice, and open-ended formatted answers. The questionnaire was subdivided into three sections: (1) demographic information (8 questions), (2) in-office COVID-19 mitigation approaches (32 questions), (3) history of COVID-19 infection and COVID-19 vaccination, and attitudes of the orthodontic providers (10 questions). The quick response (QR) code was generated to link the questionnaire (Fig. 1)

### Statistical analysis

Descriptive statistics analysis by frequencies (%) were performed for each of the survey questions along with selected cross tabulations. When applicable, Chi-square statistics for associations were assessed. Statistical significance was set at *α* = 0.05 level. The data was analyzed using IBM SPSS Statistics for Windows, version 28.0.

## Results

### Participants

**Fig. 2:**
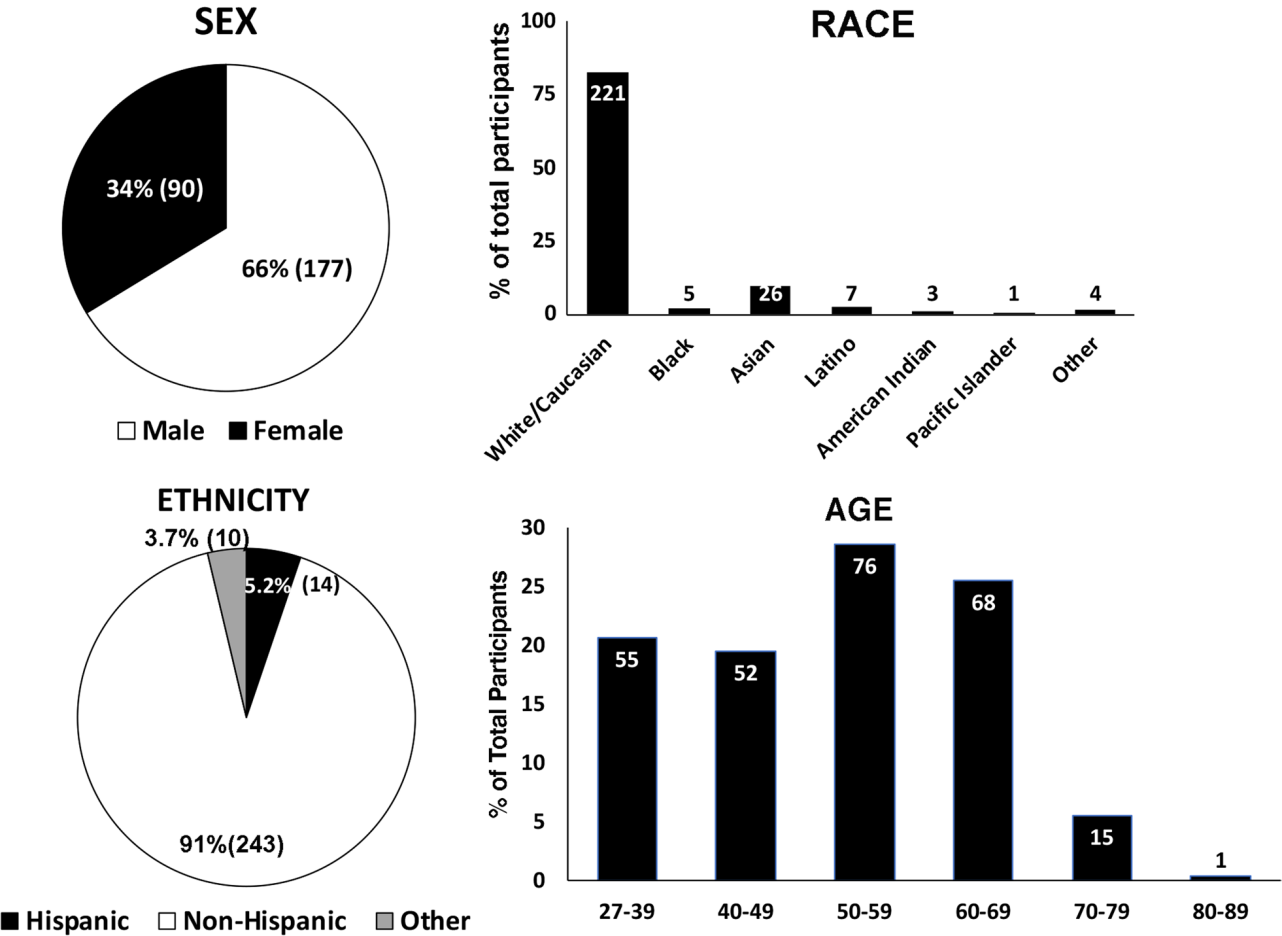
Biological demographic profiles of the participants. The demographic profile represents orthodontic providers in all geographical areas in the United States

**Fig. 3:**
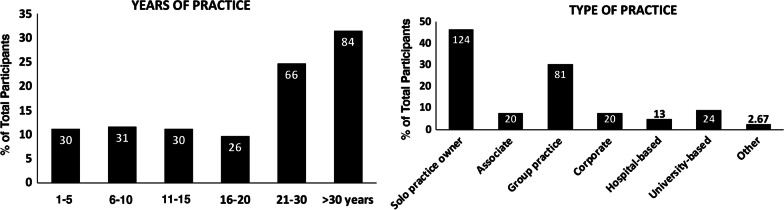
Practice demographic profiles of the participants. The demographic profile represents orthodontic practice locations in all geographical areas in the United States

This cross-sectional design study was disseminated to 12,393 practitioners, and 457 returned responses (response rate 3.69%) from January 1st, 2021 to December 31st, 2021. We observe that with the resulting sample size we can estimate all proportions to within 6% points with a confidence level of at least 95%. Of those responses, 154 were from pediatric dentists who did not provide orthodontic treatment and the data from this group were excluded from the analysis. About 66.3% of respondents were identified as males and 33.7% as females. About 82.8% of respondents were white/Caucasian, with the second-largest population being Asian (9.7%). About 91.0% of respondents were ethnically non-Hispanic (Fig. 2). The primary group of respondents was aged 50–59 (28.5%) and followed by the group of age 60–69 (25.5%) (Fig. 2). Over 50% of respondents have been practicing for at least 21 years. About 46.4% of respondents were identified as solo practitioners, while 30.3% responded that he or she was in a group practice setting. 7.5% were associated, 7.5% worked in a corporate office, 4.9% were hospital-based, and 9.0% were university-based. 2.6% of respondents listed “other” practice types, including military service and working at federally qualified health centers (FQHCs), as shown in Fig. 3. The respondents were distributed into geographical regions by AAO constituencies using their zip codes. The largest group of respondents was located in the states represented by the Midwest Society of Orthodontists (85 responses, 31.8%). The second-highest respondent group was located in the Southern Society of Orthodontists states (44 responses, 16*.5*%), as displayed in Table 1.

**Table 1:** Geographic distribution of respondents

Society of orthodontists	Percent (%)
Midwestern	31.8
Southwestern	8.2
Southern	16.5
Pacific Southwest	11.6
Middle Atlantic	7.9
Northeastern	10.9
Great Lakes	9.7
Rocky Mountain	3.4
Total	100

### Landscape of orthodontic practice management

**Fig. 4:**
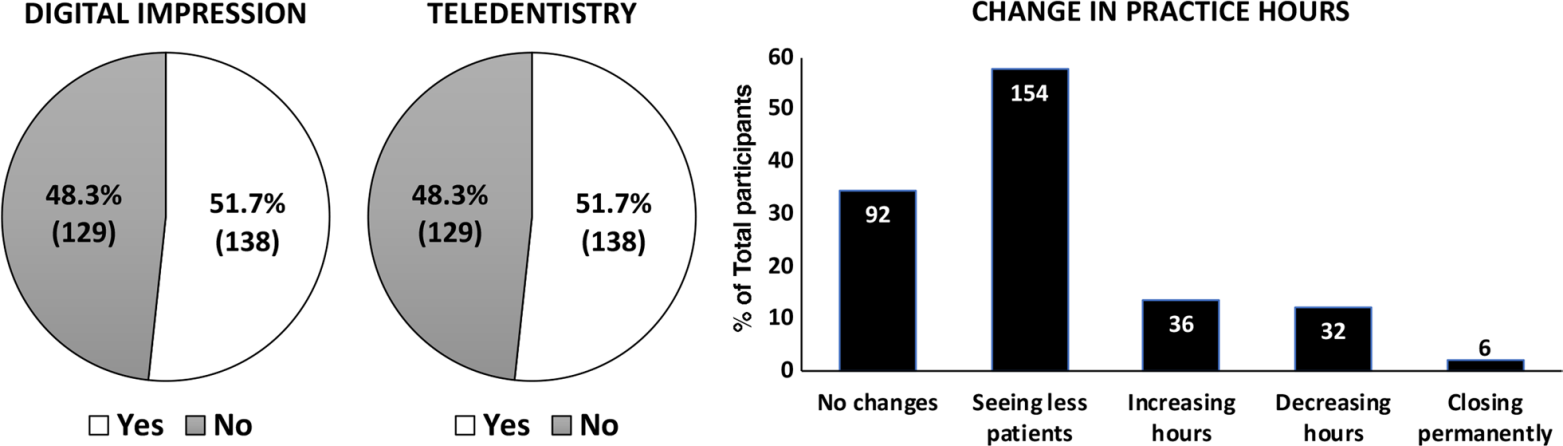
Changes of clinical practice management due to COVID-19 pandemic. The representatives of changes in contemporary orthodontic practice affected by the COVID-19 pandemic

According to the respondents, if they offered teledentistry to their patients after the pandemic, 138 respondents reported “yes” (51.7%), and the same number of respondents switched to digital impressions instead of alginate impressions to prevent the spread of COVID-19 (Fig. 4). Regarding practice-hour changes in response to the COVID-19 pandemic, 57.7% reported seeing fewer patients, while 12.0% reported decreased working hours and 13.5% reported increasing working hours. Six respondents (2.2%) closed their practices permanently (Fig. 4). The most noticeable group that reported seeing fewer patients was in the > 30 years practicing group, in which 59 out of 84 respondents (70.2%) reported seeing fewer patients.

### Infection controls

According to the respondents, the most common sources of information regarding COVID-19 infection control were the American Dental Association (80.9%), the US Centers for Disease Control and Prevention (CDC) (74.2%), State Dental Association (58.1%), and American Association of Orthodontists (AAO) (49.4%). Respondents were able to select more than one option. A frequency summary of information sources used is displayed in Table 2. Almost all practitioners reported disinfecting commonly touched surfaces and equipment in operatories between patients and offered staff facemasks. Most respondents required social distancing in the treatment area (206, 77.2%) and air purifiers or other filtration systems (180, 67.4%). Other patient pretreatment screening for infection control efforts included having patients fill out an exposure risk questionnaire (193, 72.3%), checking temperatures of both patients and self/staff (189, 70.8% and 170, 63.7%), and having parents or guardians wait outside the practice during the appointment (134, 50.2%). A complete summary of infection control results is included in Table 3. Regarding any struggles to attain PPE for their offices, 167 respondents (62.5%) reported “yes”. About 115 (43.1%) cited increased costs of PPE, 166 (62.2%) cited limited supplies, 69 (25.8%) described the lower quality of PPE products. There were no statistically significant associations between geographic location and difficulty acquiring PPE, indicating that providers had difficulty acquiring PPE nationwide. However, there was a statistical significance of the association between the experience and reporting difficulties acquiring PPE (Chi-square value = 15.133, p = .010). As practice experience increased, more respondents reported PPE acquisition difficulties.

**Table 2:** Reported source of information regarding COVID-19 (responders could choose more than one)

Source of information	Percent (%)
American dental association (ADA)	80.8
American association of orthodontists (AAO)	49.4
American academy of pediatric dentistry (AAPD)	29.2
Occupation safety and health administration (OSHA)	43.8
Organization for safety and aseptic procedures (OSAP)	2.6
State dental association	43.0
Local health department	32.9
Centers for disease control and prevention (CDC)	74.1
World health organization (WHO)	9.7
Dental school website	6.3
Other	5.2

**Table 3:** Reported infection control measures

Infection control effort	Percent (%)
High-powered Suction Modification (i.e., Isolite or Dryshield)	26.2
Extraoral high-power suction	31.1
Installed physical barriers (between units)	40.1
Installed physical barriers (between patients and doctors)	7.5
UV light systems	19.5
Air purifiers or other filter systems	67.4
Negative pressure room	7.1
Exposure risk questionnaire	72.3
Pre-visit screening (1–2 days prior)	53.2
Temperature check (patient)	70.8
Temperature check (self/staff)	63.7
Pre-treatment rinse	39.0
Disinfect frequently touched surfaces	99.6
Social distancing (treatment area)	77.2
Patients waiting outside the practice	50.2
Parent/guardian allowed in treatment area	69.3
Disinfect all equipment in operatory	98.9
Provide facemasks (staff)	99.6
Provide facemasks (patients)	70.8

### Covid-19 infection and transmission

Regarding the history of COVID-19 infection of the providers and their staff members, 16 doctors (6.0%) responded “yes”. One practitioner (6.3%) speculated a staff as the origin of transmission, one practitioner (6.3%) speculated a patient, and 14 practitioners (87.5%) speculated sources from outside the office. Regarding the history of COVID-19 of the staff members, 184 (68.9%) responded “yes”, with 17.4% respondents having one staff member test positive, 23.4% having two staff members test positive, 19.0% having three staff members test positive, and 40.2% having more than three staff members test positive. Regarding transmission sources, 2.7% reported possible in-office transmission, 1.1% reported possible transmission from patients, and 96.2% reported possible transmission from sources outside their practices. 256 out of 267 respondents provided their staff with workplace guidelines for COVID-19 transmission and exposure prevention.

### COVID-19 vaccination status

Regarding COVID-19 vaccination status, 94.0% replied “yes”, Twelve practitioners (4.5%) responded “no” to receiving the vaccine and the remaining did not state in the response. The reason for the COVID-19 vaccine hesitancy in the providers is summarized in Table 4. Regarding the COVID-19 vaccination status in their staff, 249 respondents (93.3%) reported that their staff had received the vaccine. Regarding the approach to encourage the COVID-19 vaccination in the staff, 72.7% reported that they would educate the staff member on the safety of the vaccine, 31.1% would refer the staff member to their primary care physician, 13.5% would use peer pressure, 23.2% would do nothing, and 13.1% stated other reasons.

**Table 4:** Orthodontic providers’ reasons for not receiving COVID-19 Vaccine (*n* = 12)

Reasons for Not Receiving Vaccine	Frequency	Percent (%)
Lack of access	1	6.7
Lack of trust	7	46.7
Previously infected with COVID-19	2	13.3
Mitigation measures are sufficient protection	4	33.3
Medical exemption	2	13.3
Religious exemption	0	0
Other	2	13.3

### Association between demographic data and COVID-19 infection control and infection

Crosstabulation statistical analyses were performed to evaluate the associations between the respondents’ age and various parameters tested in our survey. There were no statistically significant associations between the respondents’ age, vaccination rate, and COVID-19 infection rate. This lack of statistical significance could be attributed to overall high vaccination rates (94.0% for doctors), lower COVID-19 infection rate (6.0%), and an overall willingness to encourage vaccination (70.8%). Crosstabulation statistical analyses were tested to evaluate the associations between the geographic location of practices and various questions. No significant associations were found in changes in practice hours, vaccination rate, difficulty in acquiring PPE, COVID infection, and willingness to encourage staff to receive the vaccine. The lack of association between vaccination rate and vaccine encouragement could be due to overall high vaccination rates and willingness to encourage vaccination. When analyzing the COVID-19 infection rate, no association could be attributed to our samples’ relatively low infection rate (16 out of 267 respondents, 6.0%).

## Discussion

This comprehensive study aimed to evaluate COVID-19 infection rates and mitigation strategies to prevent the transmission of COVID-19 in orthodontic provider-specific settings. The participants’ demographic profiles in this study represent 84.8% private practitioners with broad geographical locations and as practice owners who represented the actual orthodontic practitioners in the United States. The overall infection rate in this study was 6.0%, which is higher than a similar longitudinal study in the general dentist population (2.6%) and the general population (1.1%) [12]. The range of response for survey research in the literature is 33–44%; however, the response rate could be varied depending on the topics, incentives and targets of participants [21, 22]. In addition, online surveys yielded an average 12% lower response rate than other modes of surveys[23]. We speculate that our low rate of participation was due to no incentives for participation and the length of questionnaires. However, the validated and comprehensive set of questionnaire in this study provides information of practice management and evidence for the orthodontic practices for the preparation of orthodontic practice for the future pandemic event. A recent report showed the positivity rate in orthodontic patients was 0.626% and a potential risk of COVID-19 transmission from patients to orthodontic providers remains, even with asymptomatic and vaccinated patients [15]. Our survey respondents cited the ADA website (80.9%), CDC website (74.2%), and state dental association websites (58.1%) as the most commonly used sources for COVID-19 information. These results are similar to a previous study in an orthodontic population, which found that 73% of respondents cited professional association websites as the most commonly accessed sources [20]. More use of social media news sources was reported in the previous study [20]. Questions 9–40 gauged practitioners’ mitigation approaches with similar questions in a previous study of general dentists [12]. Regarding wearing masks/eye protection, our results were consistent with a similar study of general dentists. 85.4% responded that they were wearing goggles or glasses, similar to the 81.8% of general dentists who reported always wearing masks and eye protection, regardless of the procedure [12]. As practice experience increased, more respondents reported PPE acquisition difficulties. This finding could be attributed to the observation that more experienced respondents often reported solo practice ownership, leaving the burden of acquiring PPE on them. In contrast, the less experienced respondents may work as associates who are not responsible for acquiring the PPEs. Disinfection of frequently touched surfaces was reported in 99.6% of our respondents (266 out of 267), similar to results found in a general dentist population (99.7%) [12]. However, our results showed lower percentages of orthodontists providing temperature screening, physical protection in the office, pre-appointment screenings, and encouraging social distancing. These results ranged from 40.1 to 77.2%, depending on the type of infection control measure. A similar study in a general dentist population showed that these measures were employed by greater than 95% of general dentists [12]. A study suggested simple screening methods are not sufficient and point-of-care (POC) testing may be implemented in dental offices [24]; however, the cost of unit and specificity and sensitivity of the tests are still controversial for routine application[25, 26].

Our results are similar to those found in a general dentist population regarding enhanced mask use; 111 out of 267 (41.6%) of respondents confirmed they were wearing an N95 respirator, while 127 out of 267 (47.6%) reported wearing a KN95. In a general dentist population, an average of 59% of respondents replied that they wore an N95 or equivalent during some procedures [12]. This study showed that most respondents used face shields and goggles or glasses (67.8% and 85.4%, respectively). These infection control measures can help mitigate the spread of COVID-19 through the prevention of eye exposure [27]. Overall, our respondents appear to be taking the necessary steps to mitigate the spread of COVID-19 in their offices through stringent disinfection and proper PPE use. Increasing the use of intraoral suction devices and pretreatment rinses should be encouraged as adjunctive steps to lower the transmission risk of COVID-19 and similar pathogens [28].

Overall, the 6% infection rate was significantly lower than that found in a similar study of frontline healthcare providers, which showed a prevalence rate of 29% [29]. This lower positivity rate in an orthodontic population could be attributed to the increased use of proper PPE throughout the pandemic as the standard infection control in dental practices to prevent SARS-CoV-2 transmission [28]. Most respondents attributed their infection sources to outside the office (87.5%). This result would also support the conclusion from the studies reporting that proper PPE in an office setting limits the transmission of COVID-19 even in a relatively high-risk setting, as the providers may not be as stringent in their PPE use outside of the office [28, 30]. The overall vaccination rate for the orthodontic providers in our study was 94.0% which was significantly higher than the rate in the general population (63.8% as of January 31st, 2022) [31]. This vaccination rate is similar to one published according to the ADA Health Policy Institute, which reported 89.8% of dentists were fully vaccinated (as of June 2021) [32] and is also significantly higher than the vaccination rate in healthcare providers (70.0% as of September 15th, 2021) [33]. A higher vaccination rate in dentists compared to other healthcare workers could be attributed to the fact that all dental procedures require the removal of a facemask with the increased risk of COVID-19 transmission. In United Kingdom, 21% of orthodontic providers were not confident about the potential beneficial effects of a vaccination programme on orthodontic clinical service provision [34]. Geographically, there was no significant difference between constituencies in the overall vaccination rate. There were reports of COVID-19 hesitancy among dentists, dental hygienists, and dental students [35–37]. However, in the orthodontic provider population in this study, the rate of vaccination in this population is relatively high. This finding is most likely attributed to a high vaccination rate in dental providers, regardless of geographic location.

### Limitations

This study has several limitations. First, the emergence of variants of SARS-CoV-2 at different duration during pandemic drove the changes in the transmission rate of COVID-19 in the population. Second, the COVID-19 infection control policy was consistently changed with the emergence of SARS-CoV-2 variants. Each SARS-CoV-2 variant possessed its transmissibility and severity of the symptoms. The infection control and patient screening approaches changed and overlapped with the announcement of the professional organization and central government policy. Third, the nature of response rate for the online questionnaire study is low; however the completeness and the cost-effectiveness of online format was higher compared to paper and pencil format. Though we sent two-time reminding emails to all participants and encouragement of confidentiality for participation, the response rate was not increased. The survey was performed as online and anonymous format to minimize desirability bias even though the participants’ bias may exist during the survey since most of orthodontic providers may have practice universal precaution to prevent cross-contamination.

## Conclusion

High percentage of orthodontic providers increased COVID-19 mitigation strategies to prevent in-office transmission and vaccinated against COVID-19. Low infection rates of COVID-19 in the orthodontic providers implicated that the implemented infection control measures successfully limited COVID-19 transmission in orthodontic practices.

## Data Availability

The datasets used and/or analyzed during the current study are available from the corresponding author on reasonable request.

## Abbreviations

COVID-19: Coronavirus disease 2019
REDCap: Research electronic data capture
SARS-CoV-2: Severe acute respiratory syndrome Coronavirus 2
CDC: Centers for disease control and prevention
PPE: Personal protective equipment
AAO: American association of orthodontists
AAPD: American academy of pediatric dentistry
ADA: American dental association
QR: Quick response
POC: Point of care
N95: Standard for United States system for mask or respirator
KN95: Standard for Chinese system for mask or respirator
